# Enhanced poly(3-hydroxypropionate) production via β-alanine pathway in recombinant *Escherichia coli*

**DOI:** 10.1371/journal.pone.0173150

**Published:** 2017-03-02

**Authors:** Stephen Tamekou Lacmata, Jules-Roger Kuiate, Yamei Ding, Mo Xian, Huizhou Liu, Thaddée Boudjeko, Xinjun Feng, Guang Zhao

**Affiliations:** 1 CAS Key Laboratory of Biobased Materials, Qingdao Institute of Bioenergy and Bioprocess Technology, Chinese Academy of Sciences, Qingdao, China; 2 Laboratory of Microbiology and Antimicrobials Substances, Department of Biochemistry, Faculty of Sciences, University of Dschang, Dschang, Cameroon; 3 Institute of Oceanology, Chinese Academy of Sciences, Qingdao, China; 4 Laboratory of Phytoptotection and Valorization of Vegetals Resources, Biotechnology-Nkolbisson Center, Department of Biochemistry, University of Yaounde, Yaounde, Cameroon; 5 Randian Technology Company Limited, Tianjin, China; Universite Paris-Sud, FRANCE

## Abstract

Poly(3-hydroxypropionate) (P3HP) is a thermoplastic with great compostability and biocompatibility, and can be produced through several biosynthetic pathways, in which the glycerol pathway achieved the highest P3HP production. However, exogenous supply of vitamin B_12_ was required to maintain the activity of glycerol dehydratase, resulting in high production cost. To avoid the addition of VB_12_, we have previously constructed a P3HP biosynthetic route with β-alanine as intermediate, and the present study aimed to improve the P3HP production of this pathway. L-aspartate decarboxylase PanD was found to be the rate-limiting enzyme in the β-alanine pathway firstly. To improve the pathway efficiency, PanD was screened from four different sources (*Escherichia coli*, *Bacillus subtilis*, *Pseudomonas fluorescens*, and *Corynebacterium glutamicum*). And PanD from *C*. *glutamicum* was found to have the highest activity, the P3HP production was improved in flask cultivation with this enzyme. To further improve the production, the host strain was screened and the culture condition was optimized. Under optimal conditions, production and content of P3HP reached to 10.2 g/L and 39.1% (wt/wt [cell dry weight]) in an aerobic fed-batch fermentation. To date, this is the highest P3HP production without VB_12_.

## Introduction

Poly(3-hydroxypropionate) (P3HP) is a promising polymer with high rigidity, ductility, and exceptional tensile strength in drawn films, and can be synthesized chemically by ring opening polymerization of β-propiolactone [1]. However, the chemical synthesis is not suitable for industrial scale production of P3HP because β-propiolactone is a human carcinogen. As 3-hydroxypropionate (3HP) is not a common metabolite in most organisms, biosynthesis of 3HP-containing polymers was usually based on structurally-related precursors, such as 3HP and acrylic acid [1], whereas the precursors are generally expensive and toxic to the cells. So P3HP biosynthesis from inexpensive carbon sources has attracted much more interest recently.

In our previous study, three strategies have been adopted for P3HP biosynthesis. One pathway starts from malonyl-CoA, which is reduced to free 3HP by the malonyl-CoA reductase (MCR) from *Chloroflexus aurantiacus*. Unfortunately, our recombinant *Escherichia coli* strain accumulated only 13 mg/L P3HP when using glucose as sole carbon source [2]. In the second strategy, glycerol is converted into 3-hydroxypropionaldehyde by glycerol dehydratase from *Klebsiella pneumoniae*, followed by CoA ligation with propionaldehyde dehydrogenase from *Salmonella typhimurium*, and polymerization with polyhydroxyalkanoate synthase from *Cupriavidus necator*. Though 10.1 g/L P3HP was produced from glycerol in fed-batch fermentation, exogenous supply of vitamin B_12_ was required to maintain the activity of glycerol dehydratase, which resulted in high production cost [3].

**Fig 1:**
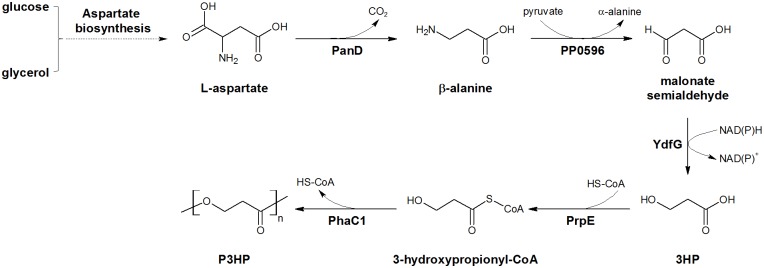
β-Alanine pathway used in this study. Four L-aspartate decarboxylases (PanD) from *E*. *coli*, *B*. *subtilis*, P. *fluorescens*, and *C*. *glutamicum* were tested. PP0596, β-alanine-pyruvate transaminase from *Pseudomonas putida*; YdfG, 3-hydroxyacid dehydrogenase from *E*. *coli*; PrpE, propionyl-CoA synthase rom *E*. *coli*; PhaC1, polyhydroxyalkanoate synthase from *Cupriavidus necator*.

Then a new pathway employing β-alanine as an intermediate was constructed (Fig 1) [4]. In this pathway, L-aspartate produced from aspartate biosynthesis pathway was converted into β-alanine using L-aspartate-α-decarboxylase (PanD) from *Escherichia coli*, β-alanine was converted into malonate semialdehyde by β-alanine-pyruvate transaminase of *Pseudomonas putida*, and then malonate semialdehyde was reduced by 3-hydroxy acid dehydrogenase from *E*. *coli* to 3-HP as precursor of P3HP. Although this pathway has some advantages such as being redox neutral and does not require VB_12_, the P3HP production and content are relatively lower than other pathways. High cost and low production seriously restricted the industrialization of P3HP. In previous study, it was noticed that supplement of β-alanine could significantly improve the P3HP production, indicating that the P3HP yield of β-alanine pathway is limited by two possible reasons: low activity of PanD or low intracellular L-aspartate concentration. Further study should be done to verify the real cause and improve the P3HP production.

This study was aimed to find the rate-limiting step of the new P3HP pathway and improve the production for industrial scale. Based on the results, firstly we figured out that insufficient β-alanine restricts the P3HP biosynthesis and poor PanD is the rate-limiting enzyme in the β-alanine pathway. Through enzyme screening, PanD from *Corynebacteria glutamicum* was proved to have higher enzyme activity and could improve the P3HP production dramatically. Under optimal conditions, the resultant *E*. *coli* strain produced 0.98 g/L and 10.2 g/L P3HP in flask cultivation and fed-batch fermentation, respectively. To our knowledge, this is the highest P3HP production without VB_12_, which is a good step for the industrialization.

## Materials and methods

### Bacterial strains and plasmid construction

The bacterial strains and plasmids used in this work are listed in Table 1. The plasmid pHP302 carrying *phaC1* and *prpE* genes [2] and plasmid pWQ513 harboring *ydfG*, *PP0596* and *panD* from *E*. *coli* [4] were constructed previously. *C*. *glutamicum* ATCC 13032 was purchased from ATCC for *panD*_*Cg*_ cloning [5]. The *panD* genes from *Bacillus subtilis* (*panD*_*Bs*_) [6] and *Pseudomonas fluorescens* (*panD*_*Pf*_) were codon-optimized according to *E*. *coli* codon preference, and synthesized by Genewiz Company (Beijing, China). The *panD*_*Ec*_ gene in pWQ513 was replaced by *panD*_*Bs*_, *panD*_*Pf*_, and *panD*_*Cg*_ to generate plasmid pFS01, pFS02, and pFS03, respectively.

**Table 1:** Bacterial strains, plasmids, and primers used in this study.

Strain, plasmid, and primers	Description	Source
Strains		
*Escherichia coli* DH5α	F^-^ *supE*44 Δ*lacU*169 (Φ80 *lacZ* Δ*M15*) *hsdR*17 *recA*1 *endA*1 *gyrA*96 *thi*-1 *relA*1	Invitrogen
*E*. *coli* JM109(DE3)	*recA1 endA1 gyrA96 thi-1 hsdR17 supE44 relA1* Δ(*lac-proAB*) [F’ *traD36 proAB*^*+*^ *lacI*^*q*^ *lacZ* ΔM15] λ(DE3)	Promega
*E*. *coli* BL21(DE3)	F^-^ *omp*T *hsd*S_B_ (r_B_^-^ m_B_^-^) *gal dcm* (DE3), source of *panD*_*Ec*_	Novagen
*E*. *coli* BL21(DE3)Star	F^-^ *omp*T *hsd*S_B_ (r_B_^-^ m_B_^-^) *gal dcm rne*131 (DE3)	Invitrogen
*E*. *coli* BL21(DE3) Rosetta	F^-^ *omp*T *hsd*S_B_ (r_B_^-^ m_B_^-^) *gal dcm* (DE3)pRARE^2^	Novagen
*Corynebacterium glutamicum* ATCC 13032	Source of *panD*_*C*_ gene	ATCC
Q2153	*E*. *coli* BL21(DE3) bearing pHP302 and pWQ513	This study
Q2542	*E*. *coli* BL21(DE3) bearing pHP302 and pFS01	This study
Q2543	*E*. *coli* BL21(DE3) bearing pHP302 and pFS02	This study
Q2544	*E*. *coli* BL21(DE3) bearing pHP302 and pFS03	This study
Q2154	*E*. *coli* BL21(DE3) Star bearing pHP302 and pFS03	This study
Q2155	*E*. *coli* BL21(DE3)Rosetta bearing pHP302 and pFS03	This study
Q2555	*E*. *coli* JM109(DE3) bearing pHP302 and pFS03	This study
Plasmids		
pHP302	rep_pBR322_ Amp^R^ lacI P_T7_ *phaC1 prpE*	[2]
pWQ513	rep_p15A_ Cm^R^ *lacI* P_T7_ *ydfG panD*_*Ec*_ *panM* P_T7_ *PP0596*	[4]
pFS01	rep_p15A_ Cm^R^ *lacI* P_T7_ *ydfG panD*_*Bs*_ *panM* P_T7_ *PP0596*	This study
pFS02	rep_p15A_ Cm^R^ *lacI* P_T7_ *ydfG panD*_*Pf*_ *panM* P_T7_ *PP0596*	This study
pFS03	rep_p15A_ Cm^R^ *lacI* P_T7_ *ydfG panD*_*Cg*_ *panM* P_T7_ *PP0596*	This study
Primers		
*panD*_*Bs*_ cloning		
1251	CCCAAGCTTAAGGAGATATACATGTACCGTACCATGATGTC	
1252	CCCAAGCTTTTACAGGATGGTACGAGC	
*panD*_*pf*_ cloning		
1253	CCCAAGCTTAAGGAGATATACATGCACGCTATCATGCTG	
1254	CCCAAGCTTTTAAGCCAGCTGAACCGGGATAG	
*panD*_*cg*_ cloning		
C-*panD*-F	CCCAAGCTTAAGGAGATATACATGCTGCGCACCATCCTCG	
1024	CCCAAGCTTCTAAATGCTTCTCGACGTC	

### Shake flask culture

The engineered strains were grown in a modified minimal medium as described previously, which contains 20 g glycerol, 3g glucose, 1.5 g KH_2_PO_4_, 3 g (NH_4_)_2_SO_4_, 1 g citric acid, 1 g citrate sodium, 1.9 g KCl, 3 g MgSO_4_, 0.138 g FeSO_4_·7H_2_O, 45 mg vitamin B_1_, and 1 ml of trace element solution per liter[3]. Shake flask cultures were carried out in 500 ml baffled flasks containing 100ml medium at 37°C in an orbital incubator and shaken at 200 rpm with an orbit diameter of 26 mm. The cells were induced with 0.05mM IPTG at OD_600_ ~ 0.6 unless specified. The precursors (β-alanine and L-aspartate) and organic nitrogen sources (tryptone, soy peptone, yeast extract and beef extract) were added as indicated in the text. The P3HP was extracted from the lyophilized cells with hot chloroform as described [7]. All shake-flask experiments were performed in triplicate.

### Fed-batch fermentation

Fed-batch fermentation were carried out in a Biostat B plus MO5L fermentor (Sartorius Stedim Biotech GmbH, Germany) under the optimal conditions obtained through shake flask experiments. 3 g/L glucose and 20 g/L glycerol was used as initial carbon sources. After the initial carbon sources were nearly exhausted, fed-batch mode was commenced by feeding a solution containing 10M glycerol. Ammonia (25% in water) was added automatically to control the pH at 7.0. The dissolved oxygen concentration was maintained at 20% saturation. At OD_600_ of 10, the cells were induced by adding 0.05mM IPTG. IPTG and antibiotics were added every 24h during 72h fermentation.

### SDS-PAGE analysis and aspartate decarboxylase activity assay

The strain Q2153, Q2542, Q2543, Q2544 and *E*. *coli* BL21(DE3) were grown in minimal medium at 37°C and induced by 0.05 mM IPTG at 30°C. The cells were harvested by centrifugation 3h after induction and lysed by sonication. The whole-cell lysate was used for SDS-PAGE. Proteins were separated in 12% acrylamide gels and visualized with Coomassie brilliant blue R250. L-Aspartate-decarboxylase (PanD) activity was assayed by evaluating the conversion of aspartate to β-alanine as determined by high-pressure liquid chromatography (HPLC) analysis [8].

### P3HP extraction and characterization

After all the fermentation process, the obtained cells were harvested by centrifugation and washed with distilled water. The cell pellets were lyophilized and the CDW was gravimetrically determined. The P3HP was extracted from the lyophilized cells with hot chloroform in a soxhlet apparatus and precipitated by the ice cold absolute ethanol as described [7]. The structure of the obtained P3HP was confirmed by NMR analysis using the Advanced III 600 NMR spectrometer (Bruker, Switzerland) as described in our previous study [9]. P3HP content was calculated using the ratio of P3HP weight to cell dry weight.

## Results and discussion

### Verification of the rate-limiting step in β-alanine pathway

**Fig 2:**
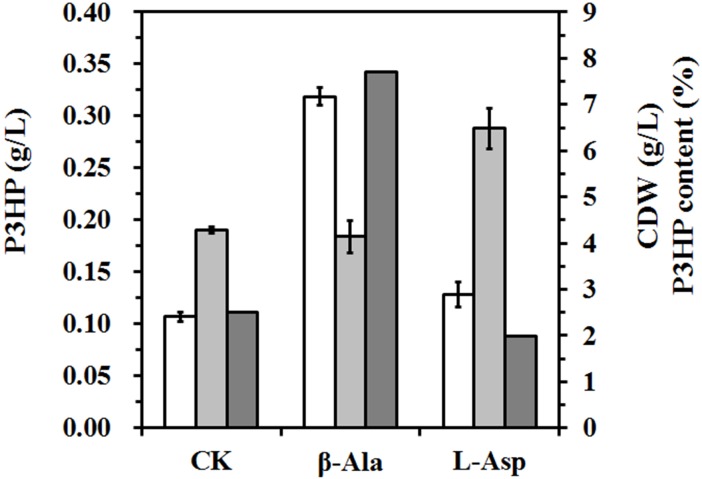
Effect of β-alanine and L-aspartate on the P3HP production. The strain Q2153 was grown in minimal medium with supplement of 5 g/L β-alanine (β-Ala) or L-aspartate (L-Asp), cultivation without amino acid was used as the control (CK), and the P3HP production (white), CDW (light grey), and P3HP content (heavy grey) were presented. The experiment was carried out in shaking flask in triplicate. All shake-flask experiments were incubated for 48 h after induction.

In previous study, we had constructed the β-alanine pathway to produce P3HP from inexpensive carbon sources [4]. The phenomenon that addition of β-alanine improved P3HP production suggested the insufficient intracellular β-alanine supply, which could be caused by two possible reasons: low activity of L-aspartate decarboxylase (PanD) or low intracellular L-aspartate concentration. To figure out the restricting factor of β-alanine pathway, *E*. *coli* BL21(DE3) strain carrying pHP302 and pWQ513 (Q2153) was grown in shake flask, and 5 g/L β-alanine or L-aspartate was added into the medium. As shown in Fig 2, the P3HP production with β-alanine addition was 0.32 ± 0.01 g/L P3HP representing 7.7% of cell dry weight (CDW), which is more than 3 times higher than that of the control group (0.11 ± 0.004 g/L), whereas L-aspartate supplement increased the cell growth dramatically and did not change much the P3HP production (0.13 ± 0.02 g/L). Also, there was a large amount of L-aspartate residues in the medium. This result demonstrates that intracellular L-aspartate cannot be converted into β-alanine effectively.

Moreover, a *panD* deleted strain lacked L-aspartate-α-decarboxylase enzyme activity and exhibited β-alanine auxotrophy [10]. And as reported, it has been suggested that the formation of β-alanine is the rate-limiting step in the pantothenate biosynthesis pathway [11], and enhanced expression of the *C*. *glutamicum panD* gene in *E*. *coli* overcomes the β-alanine limitation in pantothenate biosynthesis [8]. So, it confirmed that PanD which converts L-aspartate into β-alanine restricts the efficiency of the pathway.

### Screening of the L-aspartate decarboxylase

**Fig 3:**
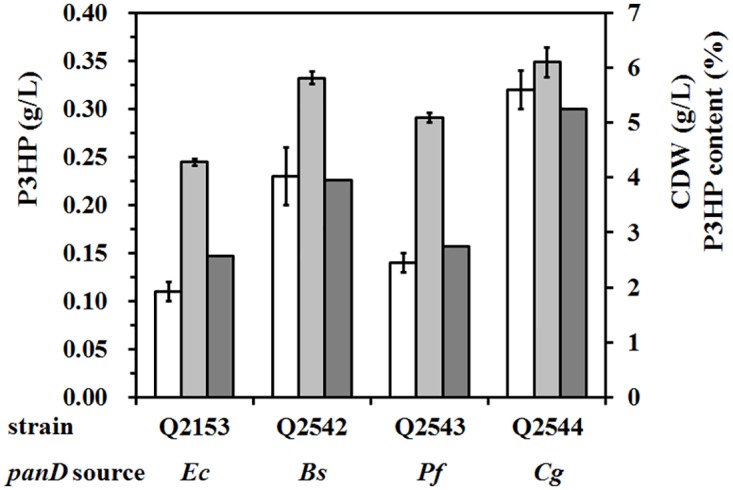
Screening of L-aspartate decarboxylase for P3HP production. P3HP-producing strains with different *panD* gene were grown in minimal medium, and the P3HP production (white), CDW (light grey), and P3HP content (heavy grey) were presented. The experiment was carried out in shaking flask in triplicate. *Ec*, *Escherichia coli*; *Bs*, *Bacillus subtilis*; *Pf*, *Pseudomonas fluorescens*; *Cg*, *Corynebacteria glutamicum*.

Science PanD was proven to be the rate-limiting enzyme, it is important to find a more efficient PanD to improve the P3HP production via β-alanine pathway. Three more genes encoding L-aspartate decarboxylase, *panD*_*Bs*_ from *B*. *subtilis* (accession No. L47709), *panD*_*Pf*_ from *P*. *fluorescens* (accession No. AY210414), and *panD*_*Cg*_ from *C*. *glutamicum* (accession No. AF116184), were cloned and used to replace the *panD*_*Ec*_ gene from *E*. *coli* in plasmid pWQ513. The resultant plasmids were transformed into BL21(DE3) strain along with pHP302 to generate P3HP-producing strains, respectively. After 48-h incubation in shake flask, the strain Q2544 carrying *panD*_*Cg*_ produced 0.32 ± 0.02 g/L P3HP (5.3% of CDW), which is 2.9-, 1.4- and 2.3-fold higher than the strains carrying *panD*_*Ec*_ (Q2153), *panD*_*Bs*_ (Q2542), and *panD*_*Pf*_ (Q2543), respectively (Fig 3), suggesting that the enzyme activity of PanD_*Cg*_ is the highest among these four enzymes. To confirm this speculation, PanD activity was assayed with crude extracts of bacterial cell cultures. The specific activity of PanD_*Ec*_, PanD_*Bs*_, PanD_*Pf*_ and PanD_*Cg*_ were 0.83 U/mg, 6.37 U/mg, 0.91 U/mg and 7.82 U/mg, respectively. SDS-PAGE was also performed to check the protein expression, however, no significant difference was observed between different PanD proteins. Furthermore, the P3HP production of strain Q2544 increased by three times when either L-aspartate or β-alanine was added into culture, indicating that PanD_*Cg*_ was sufficient to convert intracellular L-aspartate into β-alanine.

L-aspartate-α-decarboxylase found in microorganisms is usually a homotetramer, it is initially translated as an inactive precursor protein (π-chain) which undergoes intramolecular self-cleavage to create its active subunit form and cofactor [12]. The *E*. *coli* PanD is an unusual enzyme in that it requires pyruvate as a covalently bound, activated by the putative acetyltransferase PanZ [13]. No over-expressing of suitable PanZ in this study might be the reason of weak *E*. *coli* PanD activity. Compared with *E*. *coli* PanD, the maturation of *C*. *glutamicum* PanD is independent of acetyl-coenzyme A sensor such as PanZ or PanM [14]. Also, the *C*. *glutamicum panD* gene is preceded by a perfect SD sequence and can be efficiently translated, resulting in higher activity [8]. However, the activity of *C*. *glutamicum* PanD in our study is much lower than published papers [8,15], which may be caused by different *C*. *glutamicum* strains and different metabolic systems.

### Comparison of P3HP production in different *E*. *coli* strains

**Fig 4:**
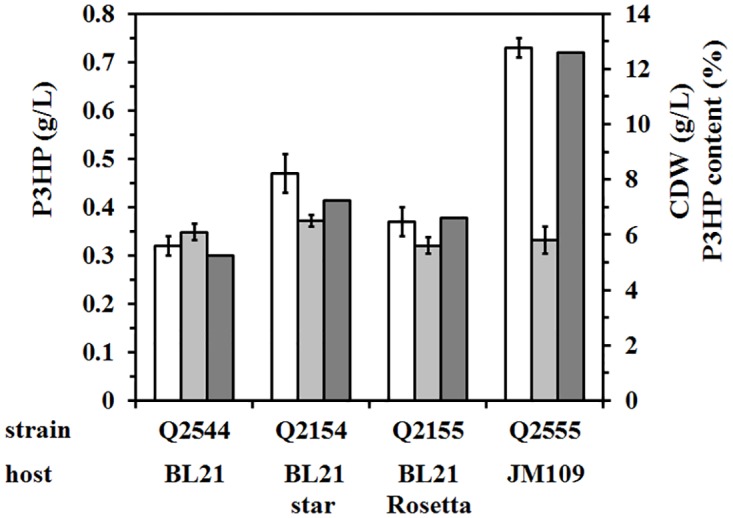
Effect of the host strain on P3HP production. Different host strains carrying plasmids pHP302 and pFS03 were grown in minimal medium, and the P3HP production (white), CDW (light grey), and P3HP content (heavy grey) were presented. The experiments were perfomed in triplicate in shake-flask cultures.

The choice of the host strain for microbial fermentation is very important for better production. To maximize the level of P3HP production, four different *E*. *coli* strains were tested here. After 48-h incubation in shake flask, the JM109(DE3) strain carrying pWQ302 and pFS03 accumulated 0.73 ± 0.02 g/L P3HP, which is much higher than the P3HP yield in BL21(DE3), BL21 star(DE3), and BL21 Rosetta(DE3), although all these four strains possessed similar cell dry weight (CDW) (Fig 4). So, JM109(DE3) strain was selected as the host for P3HP production thereafter.

### Optimisation of culture conditions

In the microbial production of desired products, culture conditions play a very important role, so optimization of culture conditions is regarded as an effective method to improve the product quality and quantity. Here, inducer concentration and organic nitrogen source were optimized to enhance P3HP yield.

In our engineered strain, the expression of heterologous genes was induced by addition of IPTG. To avoid the insufficient enzyme synthesis or metabolic burden to the cells caused by improper inducing methods, several IPTG concentrations at different time intervals were tested in shake flask culture. Addition of 0.025 mM IPTG resulted in quite low P3HP production. With increased inducer concentration, the P3HP production was remarkably improved and the maximum production of 0.98 ± 0.02 g/L was presented when IPTG was added at 0.05 mM every 24 hours (Table 2). However, IPTG at 0.1 mM severely repressed the cell growth and P3HP accumulation probably because of its cell toxicity [16].

**Table 2:** Effect of IPTG on the biomass and P3HP production.

Experiments	IPTG (mM)	Methods	CDW (g/L)	P3HP (g/L)	Content (%)
1	0.025	Once	6.67 ± 0.13	0.17 ± 0.01	2.55
2	0.025	Every 24h*	6.71 ± 0.15	0.39 ± 0.06	5.81
3	0.025	Every 12h**	5.68 ± 0.20	0.34 ± 0.01	5.99
4	0.05	Once	7.51 ± 0.37	0.73 ± 0.05	9.72
5	0.05	Every 24h*	7.01 ± 0.12	0.98 ± 0.02	13.98
6	0.05	Every 12h**	5.63 ± 0.14	0.97 ± 0.07	17.23
7	0.10	Once	4.46 ± 0.28	0.66 ± 0.03	14.80
8	0.10	Every 24h*	4.05 ± 0.09	0.71 ± 0.10	17.53
9	0.10	Every 12h**	2.95 ± 0.08	0.52 ± 0.04	17.63

*supplemented at 24h after the first time,

** supplemented at 12h, 24h and 36h after the first time

**Fig 5:**
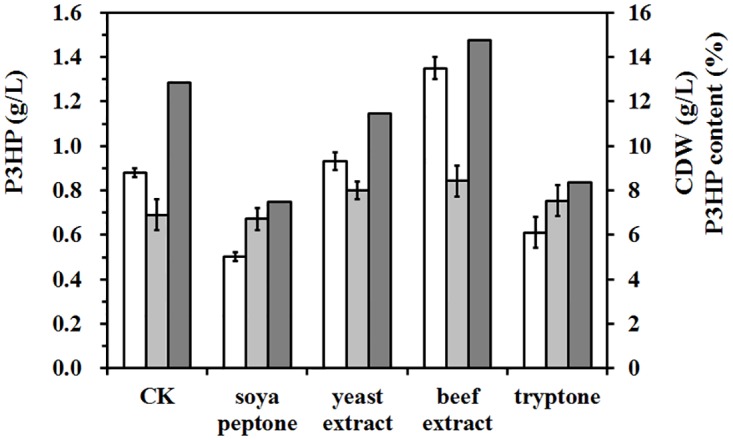
Effect of organic nitrogen on P3HP production. The strain *E*. *coli* JM109(DE3) harboring pHP302 and pFS03 was used, and 3 g/L of soya peptone, yeast extract, beef extract, and tryptone was added into the culture, respectively. Incubation without organic nitrogen was used as the control (CK). The P3HP production (white), CDW (light grey), and P3HP content (heavy grey) were presented. All shake-flask experiments were incubated at 30°C for 48 h after the first time of induction. The experiments were performed in triplicate shake-flask cultures.

To test whether addition of organic nitrogen source improves the P3HP production, 3 g/L of soya peptone, yeast extract, beef extract, and tryptone was added into the culture, respectively. Addition of beef extract increased the P3HP yield to 1.35 ± 0.07 g/L, about 1.5-times higher than the control group, whereas the P3HP production was repressed when soya peptone or tryptone was supplied in the cell culture (Fig 5). Often the PHA production can be improved under nitrogen-limited condition [17], which was also observed in this study when using soya peptone and tryptone. However, the P3HP production increased with beef extract, which is a new interesting phenomenon found in this study. The pathway used in this study work with amino acid and transaminases, so it is possible that beef extract not only provide some vitamins but also serve as a potential source of amino acid (alanine or aspartate) to enhance the efficiency of the pathway.

### Fed-batch fermentation

**Fig 6:**
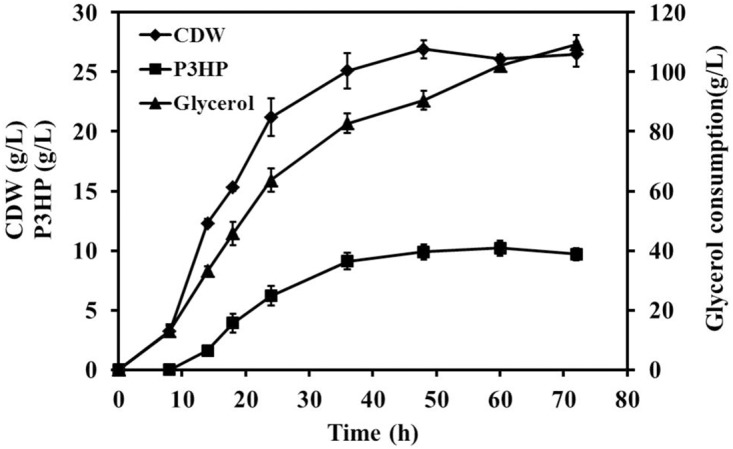
Time profiles for CDW, P3HP production and substrate consumption during an aerobic fed-batch fermentation of *E*. *coli* JM109(DE3)/pHP302/pFS03. The content of P3HP was calculated using the ratio of P3HP weight to cell dry weight.

In order to test the suitability of the recombinant *E*. *coli* strains for an improved P3HP production process, we carried out fed-batch fermentation based on the results obtained with flask cultures. The fermentations were performed under aerobic condition using *E*. *coli* JM109(DE3) harboring pHP302 and pFS03, and cell growth and P3HP accumulation were monitored over the course of fermentation. As shown in Fig 6, the cell mass reached the maximum of 26.9 g/L after 48h fermentation whereas the highest P3HP production of 10.2 ± 0.64 g/L (39.1% of CDW) was achieved after 60h. To date, this is the highest P3HP production via β-alanine pathway. However, the yield was relatively low (S1 Fig), which means that further research of the metabolism characteristics of the whole pathway should be done to improve the conversion efficiency.

Though the P3HP production in this study is a little lower compared with the previous report [18], the pathway used in this study has some advantages including no addition of coenzyme, redox neutral, and wide range of carbon sources [4]. As the bioreactor mixing efficiency may affect the cell population behavior [19] and improve the production, it is worthy to note that higher P3HP content may be obtained with optimized aeration condition, oxygen pressure, substrate addition strategy, and reactor configuration.

## Conclusions

In this study, P3HP production was improved by optimized β-alanine pathway with L-aspartate decarboxylase from *Corynebacteria glutamicum*. When PanD_C_ was overexpressed in engineered *E*. *coli* JM109(DE3) strain, 0.98 g/L P3HP was accumulated in flask cultures with optimized conditions, and the P3HP production and content reached to 10.2g/L and 39.1% (wt/wt [cell dry weight]) respectively in fed-batch fermentation with minimal medium. This is the highest P3HP production by recombinant *Escherichia coli* strain without VB_12_.

## Supporting information

S1 Fig The productivity and yield of P3HP in fed-batch fermentation. (TIF)
