# Correction: Enhanced poly(3-hydroxypropionate) production via β-alanine pathway in recombinant *Escherichia coli*

**DOI:** 10.1371/journal.pone.0174258

**Published:** 2017-03-14

**Authors:** The PLOS ONE Staff

The following information is missing from the Funding section: This study was supported by Scientific and Technological Project of Tianjin grant 14ZCZDSY00047 to GZ. The publisher apologizes for the error.
