# Supplementary figures and images for: Enhanced poly(3-hydroxypropionate) production via β-alanine pathway in recombinant Escherichia coli
Source: PLoS One. 2017 Mar 2;12(3):e0173150. doi: 10.1371/journal.pone.0173150 (PMC5333900; doi:10.1371/journal.pone.0173150)

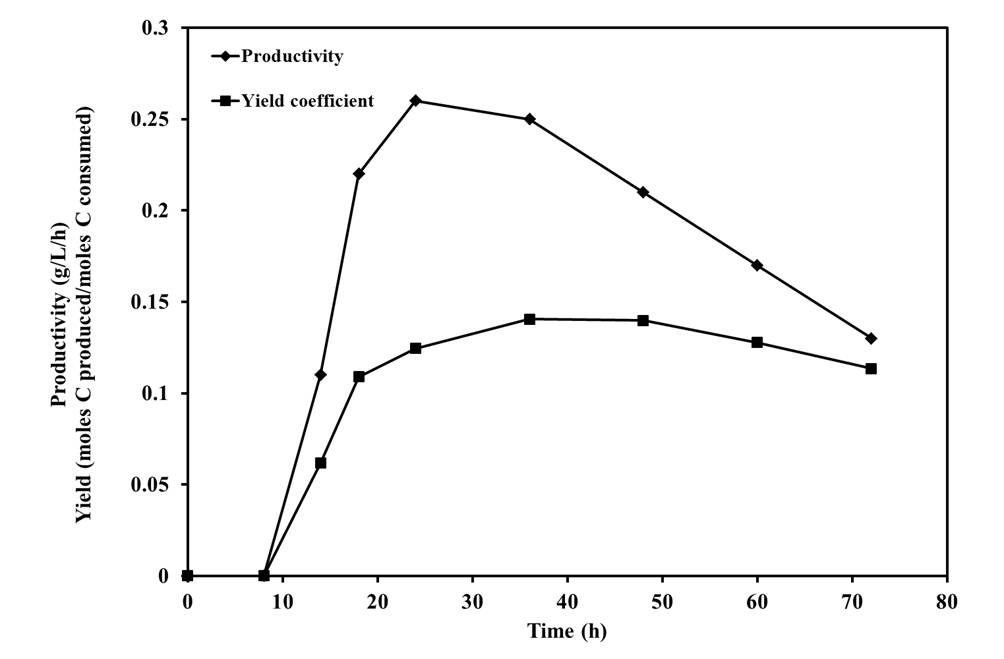

Supplement: S1 Fig — (TIF) [file pone.0173150.s001.tif]
